# Orienting asymmetries and lateralized processing of sounds in humans

**DOI:** 10.1186/1471-2202-10-14

**Published:** 2009-02-24

**Authors:** Julia Fischer, Christoph Teufel, Matthis Drolet, Annika Patzelt, Rudolf Rübsamen, D Yves von Cramon, Ricarda I Schubotz

**Affiliations:** 1 Research Group Cognitive Ethology, German Primate Center and University of Göttingen, Kellnerweg 4, 37077 Göttingen, Germany; 2 Department of Experimental Psychology, University of Cambridge, Cambridge, UK; 3 Institute of Biology-II, University of Leipzig, Germany; 4 Max Planck Institute for Human Cognitive and Brain Sciences, Department of Neurology, Leipzig, Germany; 5 Max Planck Institute for Neurological Research, Motor Cognition Group, Köln, Germany

## Abstract

**Background:**

Lateralized processing of speech is a well studied phenomenon in humans. Both anatomical and neurophysiological studies support the view that nonhuman primates and other animal species also reveal hemispheric differences in areas involved in sound processing. In recent years, an increasing number of studies on a range of taxa have employed an orienting paradigm to investigate lateralized acoustic processing. In this paradigm, sounds are played directly from behind and the direction of turn is recorded. This assay rests on the assumption that a hemispheric asymmetry in processing is coupled to an orienting bias towards the contralateral side. To examine this largely untested assumption, speech stimuli as well as artificial sounds were presented to 224 right-handed human subjects shopping in supermarkets in Germany and in the UK. To verify the lateralized processing of the speech stimuli, we additionally assessed the brain activation in response to presentation of the different stimuli using functional magnetic resonance imaging (fMRI).

**Results:**

In the naturalistic behavioural experiments, there was no difference in orienting behaviour in relation to the stimulus material (speech, artificial sounds). Contrary to our predictions, subjects revealed a significant left bias, irrespective of the sound category. This left bias was slightly but not significantly stronger in German subjects. The fMRI experiments confirmed that the speech stimuli evoked a significant left lateralized activation in BA44 compared to the artificial sounds.

**Conclusion:**

These findings suggest that in adult humans, orienting biases are not necessarily coupled with lateralized processing of acoustic stimuli. Our results – as well as the inconsistent orienting biases found in different animal species – suggest that the orienting assay should be used with caution. Apparently, attention biases, experience, and experimental conditions may all affect head turning responses. Because of the complexity of the interaction of factors, the use of the orienting assay to determine lateralized processing of sound stimuli is discouraged.

## Background

The French neuroanatomist and anthropologist Paul Broca was the first to present evidence that the circuits involved in speech production could be localized in a relatively distinct area [1]. Broca's findings also pointed towards a link between speech impairment and damage to the left side of the brain, and in the meantime, the observation of left hemispheric dominance in both production and perception of the semantic and syntactical aspects, and a right hemisphere dominance for the processing of prosodic and emotional aspects of speech, has attained the status of a firmly supported scientific finding [2,3]. More recently, comparative research has shown that lateralized processing of acoustic stimuli is not restricted to humans [4-6]. Most important for the current topic, a number of studies involving neuroanatomical, physiological and behavioural methods revealed hemispheric lateralization in the processing of species-specific sounds in non-human primates analogous to the processing of speech stimuli in humans (for a review see [5]). Japanese macaques, *Macaca fuscata*, for instance, revealed a right ear advantage – corresponding to a left hemispheric dominance – when they were trained to discriminate communicatively important features in their species-specific sounds [7]. After lesioning of the left superior temporal gyrus (STG), members of the same species showed a stronger postoperative decrement in the discrimination of species-specific calls than after an equivalent lesioning of the right STG. Finally, measuring local cerebral metabolic activity to directly detect auditory processing asymmetries in rhesus monkeys (*M. mulatta*), Poremba and colleagues [8] found a greater amount of activity in the left temporal pole in response to species-specific calls as opposed to any other sounds (see also [8] for a critical expansion).

However, many of these methodological approaches are either invasive or require extensive training of the subjects. Therefore, a simple assay introduced by Hauser and Andersson [9] has gained considerable attention and led to a series of follow-up studies in other species. This assay was first used on a population of free-ranging rhesus monkeys living on the island of Cayo Santiago. In this paradigm, a speaker was hidden directly behind subjects that were sitting in front of a food dispenser. When the animals were aligned with the speaker, either a species-specific vocalization or a bird alarm call was played and the direction of the head-turn response (with the right ear or left ear leading) was recorded. In the following, we will refer to this experimental design as the 'head turning paradigm'. These experiments and a number of follow-up studies revealed that the monkeys preferentially turned with their right ear leading when exposed to species-specific calls, and with their left ear leading in response to hetero-specific or manipulated species-specific calls [9-12]. This finding was interpreted as a demonstration of a left-hemispheric processing bias for species-specific vocalisations and a right-hemispheric bias for hetero-specific calls. While a left-hemispheric dominance for processing of species-specific calls is in line with the majority of neurophysiological and neuroanatomical research, none of these studies showed a right-hemispheric dominance for hetero-specific sounds (but see [13,14]).

More importantly, a number of recent studies employing the head turning paradigm in other species suggested a more complex pattern (see [15] for a review). Vervet monkeys, *Chlorocebus aethiops*, for example, were reported to have a left-turning (right-hemispheric) bias for processing species-specifics' calls [16] and no bias for hetero-specific sounds, while neither Barbary macaques, *M. sylvanus*, [15] nor mouse lemurs, *Microcebus murinus*, [17] turned preferentially with either their right or left ear leading in response to playback of species-specific or hetero-specific sounds. Interestingly though, the mouse lemurs showed sex differences in orienting behaviour, but only in response to specific calls [17]. Finally, analogous to rhesus monkeys, California sea lions, *Zalophus californianus*, turned preferentially with their right ear leading in response to species-specific calls but by contrast to rhesus monkeys showed no bias in response to hetero-specific calls [18], while dogs turned with their right ear leading to conspecifics' vocalizations and with their left ear leading when presented with thunder [19] in an experimental setting where these sounds were played from both sides simultaneously.

The inconsistent pattern of findings using the orienting paradigm in different species might be a result of an unexpectedly complex pattern of the phylogenetic distribution of hemispheric lateralization in closely related species. Alternatively, these findings might point towards methodological problems of the orienting paradigm, a possibility that appears to be supported by within-species inconsistencies between the orienting paradigm and more established measures of lateralized acoustic processing (see [15] for a detailed discussion). It thus seems vital to substantiate the connection between turning bias and lateralised processing before the orienting paradigm can be used to explore brain lateralization.

The purpose of the present study was to examine the link between orienting asymmetries and hemispheric lateralization in the processing of sounds in adult humans. Humans constitute an ideal test case as the lateralized processing of speech is firmly established (see above). To our knowledge, this is the first study that systematically scrutinises whether such lateralized processing indeed leads to reliable orienting asymmetries, as postulated by previous studies in nonhuman primates and other animals. To examine this link, we combined naturalistic behavioural experiments with neuroimaging techniques. The behavioural experiments were conducted in a fashion that approximated the test conditions used in the animal studies. That is, each subject was tested only once in a 'real life' situation where the subject was unaware of the purpose and the occurrence of the experiment. Speech stimuli and artificial sounds were played directly behind subjects shopping in a supermarket. The first set of these experiments was conducted in Germany. In light of the results (see below), we ran a second set of experiments in the UK to explore the possible influence of experience in a left vs. a right driving country. Functional magnetic resonance imaging (fMRI) was used on a separate population in Germany to validate the lateralized processing of the Speech stimuli employed in the naturalistic experiment in a German population.

Based on previous research, we expected a left hemispheric dominance for the processing of the speech stimuli and bilateral activation for the artificial sounds in the fMRI experiments. In accordance with the underlying assumptions of the orienting paradigm, and the left-hemisphere dominance for speech processing, we predicted that (right-handed) subjects would preferentially turn with the right ear leading when presented with human speech sounds, while they should reveal no bias in response to the presentation of artificial sounds.

Although the fMRI data confirmed the assumption of stronger left hemisphere activation for the speech sounds and bilateral activation for artificial sounds, the naturalistic behavioural experiments revealed no difference in orienting behaviour in relation to the stimulus category (Speech vs. Artificial sounds). Overall, subjects turned left more frequently, and this tendency was slightly, but not significantly stronger in German subjects.

## Results

### Behavioural data

**Figure 1:**
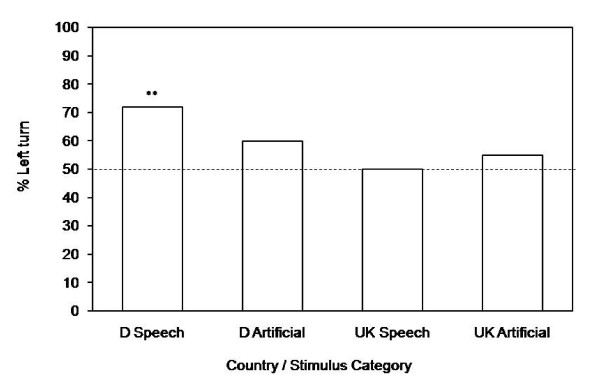
**Orienting biases in head turning experiment**. Percent left turns in response to playback of Speech and Artificial sounds in Germany and the UK, respectively. In response to speech sounds, German subjects revealed a significant left bias (* corrected p = 0.004). Orienting responses in the other three conditions were not significantly different from chance.

For the German sample, significantly more people turned left than right after playback of the speech sounds (Figure 1; binomial test, N = 61, corrected p* = 0.004; see also Table 1). Upon presentation of the artificial sounds, the tendency to turn left did not reach significance (binomial test, N = 63, p* = 0.390). There was no significant difference between the two conditions in the propensity to turn left (Fisher's exact test, N = 124, χ^2 ^= 1.931, df = 1, p = 0.187). To examine a possible influence of experience on orienting behaviour in a left vs. a right-driving country, we initiated a second set of experiments in the UK. We used the same artificial sounds, but English speech stimuli. Subjects revealed no significant orienting bias in either of the conditions (Figure 1; binomial test, N = 40 for Speech and N = 60 for Artificial, both p* = 1.0). A multinomial logistic regression with the factors stimulus category, country and gender revealed a slight but not significant influence of the country where the experiment was conducted, no other significant effects and no significant interactions (all p for interactions > 0.2; Table 2).

**Table 1:** Orienting responses in relation to stimulus category and country

**Country**	**Stimulus**	**Left**	**Right**	**Total**
D	Speech	44	17	61
	
	Artificial	38	25	63

UK	Speech	20	20	40
	
	Artificial	33	27	60

**Table 2:** Likelihood ratio tests and parameter estimates for main effects on orienting behaviour

**Factor**	**-2 LL**	**$\chi_{1}^{2}$**	**p**	**Wald**	**B**	**s.e.**
Stimulus Category	0.50	1	0.479	0.500	-0.198	0.280

Country	3.44	1	0.064	3.426	0.516	0.279

Gender	2.07	1	0.150	2.080	0.426	0.295

Results are based on a multinomial regression. Since there were no significant interactions, these were removed from the final model. -2 LL = -2 log-likelihood

These results stand in stark contrast to our prediction of a right turning bias in response to speech that was based on the assumption of a specific coupling of lateralized processing of stimuli and a turning bias. To corroborate the lateralized processing of the sound stimuli used in the experiments, we therefore used functional magnetic resonance imaging to assess the processing of the sounds. For logistic reasons, we restricted this analysis to a comparison of the German Speech sounds vs. the Artificial sounds in a German study population.

### FMRI Experiment

#### Behavioural Performance

We presented different sounds in a (sham) sound localization task in which participants were asked to judge whether the sound was presented "from the left or right" by manipulating inter-aural time differences (ITDs). Our analysis was restricted to those trials where sounds revealed no ITD (and thus simulated a midline sound-source or 0° position). Subjects were naïve to the fact that such trials also occurred.

Responses to auditory stimuli presented from the 0° position were slightly biased towards the right in both conditions (56.3% Speech, 57.6% Artificial), but these biases were not significantly different from chance: for Speech, 14 subjects showed a right (R) bias, seven a left (L) bias, and one subject no bias (binomial test N = 21, p = 0.189); in the Artificial condition, 14 subjects showed a R bias, five a L bias, and three no bias (N = 21, p = 0.064). A repeated-measured ANOVA with the within-subject factor stimulus and the between-subjects factor Gender showed no main effect of the Stimulus category (F(1, 18) = 0.073, p = 0.974), no influence of Gender (p = 0.6) and no significant interaction between Stimulus × Gender (p = 0.506).

**Figure 2:**
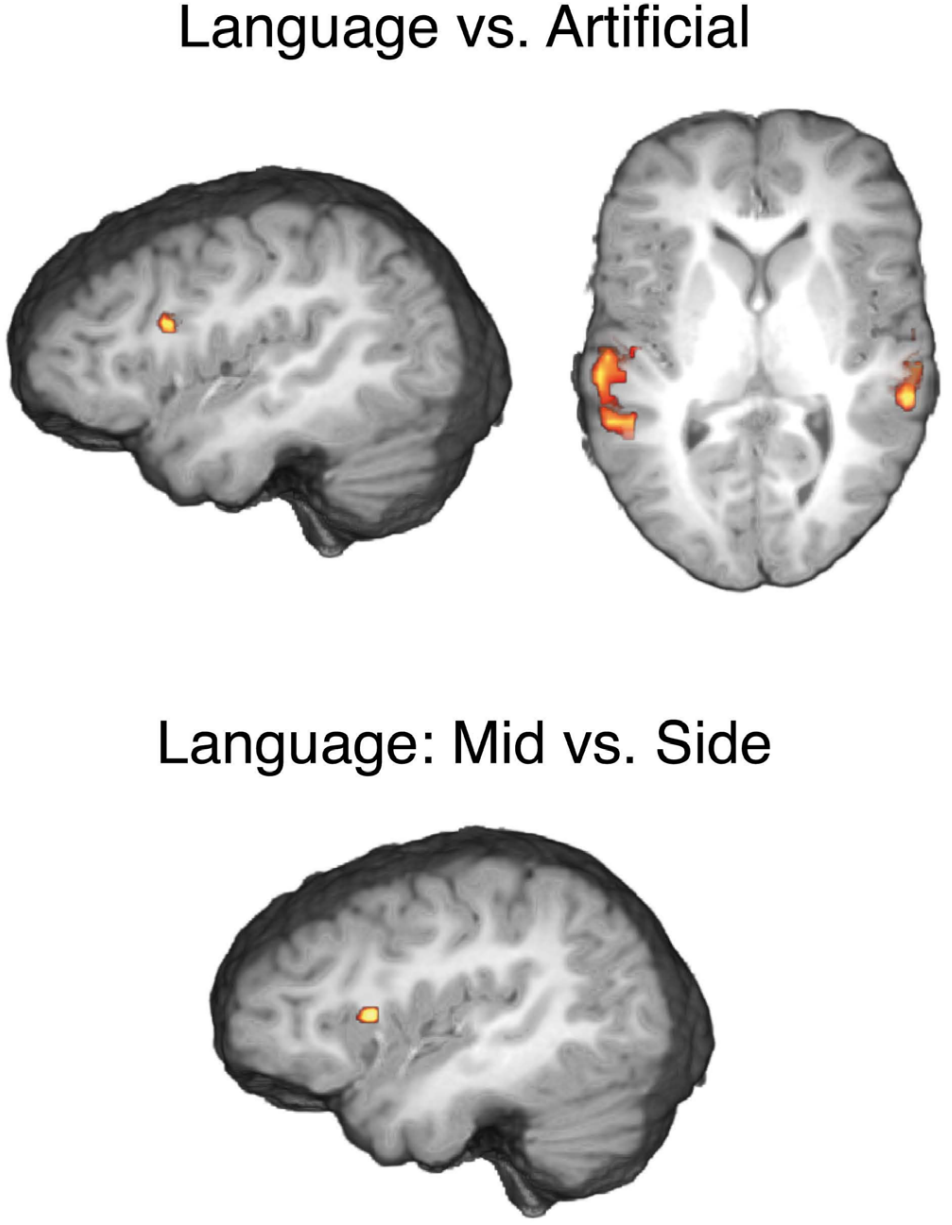
**Activation patterns in the fMRI Experiment**. Group-averaged (n = 22) statistical maps of significantly activated areas. Direct task contrasts revealed stimulus specific activations for Speech as compared to Artificial sounds in Broca's Area and in both auditory cortices. For sounds presented from the 0° position as compared to those presented from 10° or 20° from the right or the left, only Speech sounds displayed activation increase in Broca's Area.

Contrasted with the Visual control condition, each auditory location task elicited overlapping activations in a network comprising superior temporal regions and the parietal operculum, with maximal activation in the left and right gyrus of Heschl. Direct contrasts revealed activations in the left Brodmann Area (BA) 44 (Broca's Area) (Talairach coordinates x/y/z = -41/16/21 with z-max 4.3, 1998 mm^3 ^= 74 voxels) and in the superior temporal gyrus bilaterally (-53/-20/0 z-max 5.0, 10746 mm^3 ^= 398 voxels and 52/-32/9 z-max 4.5, 5670 mm^3 ^= 210 voxels) for Speech in contrast to Artificial sounds (Figure 2). When contrasting Speech stimuli virtually presented 0° from behind with those virtually presented 10° or 20° from behind, activation was found in Broca's Area (-42/13/12, z-max 4.6, 1944 mm^3 ^= 72 voxels). This effect was exclusively found for Speech sounds.

## Discussion

Contrary to our predictions, we found no significant difference of the orienting behaviour in relation to the stimulus category despite the fact that we did observe differential lateralized brain activation as a response to the different stimuli. Specifically, the left BA 44 (Broca's area) and frontal operculum showed a higher activation for Speech in contrast to Artificial sounds. In combination with the naturalistic behavioural experiments, these results indicate that in humans, hemispheric lateralization in the processing of sounds is not necessarily linked to a preferential contra-lateral orienting response.

Across the categories, subjects in the naturalistic behavioural experiment revealed a left turning bias. This bias was more pronounced in the German population, and attained significance in response to Speech stimuli. The source of this strong tendency to turn left after presentation of the speech sounds and the tendency of the English sample to show a weaker left turning bias is presently unclear. One possibility is that the orienting behaviour is governed by a tendency to shift attention to the left side in spatial tasks – in line with the assumption of a right hemisphere dominance in this domain [20,21]. On the other hand, when participants were instructed to attend to the sound source, they revealed a bias to attribute sounds coming from the 0° position as coming from the right, suggesting that spatial orientation is modulated by context [22-26]. Further behavioural experiments [see Additional file 1] suggest that this tendency is not restricted to the experimental situation but related to the instruction to localize the sound source. Thus, the results suggest that orienting responses can also be affected by the specific demands of the task employed. For practical reasons, we had to rely on self-report data regarding handedness. Obviously, this may introduce a certain degree of error, since different assessments of handedness may come to different conclusions [27]. If lateralized processing of speech, however, would lead to an asymmetry in orienting behavior equivalent to that found in some of the animal studies (> 75% in rhesus monkeys, [9]), we should still have seen an effect. This was not the case.

The fMRI data clearly demonstrated a lateralized processing of the speech stimuli used in the experiments. As an exclusive finding for Speech sounds, activity in Broca's Area was further boosted when Speech sounds were presented directly from behind. We take this to reflect a differential processing of the stimulus material in the different conditions and a further evidence for lateralized brain responses to speech. Obviously, the experimental conditions in the naturalistic setting differed from the situation during image acquisition in the scanner, where subjects were instructed to pay attention to a particular feature (sound location) and where they were exposed to repeated presentations. Both experimental modifications were deemed necessary to allow for the acquisition of imaging data without running the danger that brain activation simply reflects the default state of brain activity [28,29]. Irrespective of these modifications, the imaging data clearly show that the Speech stimuli used in the experiments evoked lateralized processing while the Artificial stimuli did not. We are thus able to rule out the possibility that the lack of stimulus-dependent orienting behaviour was due to a lack of lateralized processing of the Speech stimuli.

Taken together, our results suggest that lateralized processing of sounds does not inevitably lead to orienting biases under experimental conditions like in this study or the other studies that employed this paradigm on (relatively) free ranging animals. The comparison of the results in the naturalistic setting and under experimental conditions suggests that the task demands may affect the orienting behaviour, and it might be possible that further factors influence orienting responses. We are also aware that our experimental situation in the supermarket was not identical to the one employed for the animals where sounds were typically presented from a hidden speaker, and not from a conspecifics carrying one. Possibly, under different experimental conditions, a weak link between lateralized processing and orienting behaviour might be detected, as is the case for the dichotomous listening paradigm. Regarding the application of the head-turning paradigm, however, the contribution of other factors may mask orienting biases related to the stimulus material renders the use of the orienting assay for determining lateralized processing of sounds questionable at best. The further use of this paradigm to uncover lateralized processing of sounds is therefore discouraged.

## Conclusion

We found no contingency between lateralized processing of sounds and orienting biases. Overall, the participants revealed a tendency to turn left in the naturalistic behavioural experiments; this tendency was slightly stronger in German subjects. A comparison with the response bias in the fMRI experiments, where subjects revealed a slight bias to the right in a sound localization task, indicates that various factors such as task instructions and the experimental setting may have an effect on orienting biases. Our results – as well as the inconsistent orienting biases found in different animal species – suggest that the orienting assay applied in naturalistic settings should be used with caution. In particular, it does not seem to be possible to infer lateralized processing from orienting biases alone [9,17,18]. Unfortunately, therefore, the further use of this paradigm to uncover hemispheric lateralization cannot be recommended.

## Methods

### Naturalistic Behavioural Experiments

#### Stimuli

We used two different types of acoustic stimuli: spoken words ('Speech') [see Additional file 2] and non-linguistic artificially produced sounds ('Artificial') [see Additional file 3]. In Germany, the recorded spoken words were "Geh" (go!), "Komm" (come!), "Rad" (wheel), and "Kinn" (chin); in the UK we used "go!", "come!", "rat", "chin" and "Kinn" (from the German stimuli because this sounded similar to 'kin'). Half of the human utterances in each category were produced by two female speakers and the other half by two male speakers. The artificial sounds consisted of a total of 12 artificial stimuli generated from natural non-biological sounds which were distorted such that they could no longer be attributed to natural events. Stimuli were taken from a subset of sounds employed in a previous study [30]. These stimuli were produced by cutting and distorting portions of longer commercial sound files, with a duration of 200 ms including 10 ms rise and 40 ms fall time. Based on the results of a subjective rating study, only sounds which were classified as being unidentifiable by fifteen subjects were used in the present study (for further details, see [30]). All auditory stimuli were normalized for sound pressure level. The average stimulus duration was 330 ± 135 ms (mean ± SD).

#### Procedure

Participants for this study were chosen opportunistically, depending on their posture. Subjects had to stand still in a straight, upright posture at the time of the trial. In addition, subjects' heads were required to be straight with respect to the body on the horizontal axis (i.e. looking neither left nor right), while they could be looking up or down (i.e. bent on the vertical axis) as this should have no effect on preference to turn either right or left. Experiments in Germany were conducted in Göttingen; those in the UK in London and Cambridge.

Two experimenters participated in each trial. The first used a PalmOS handheld (Tungsten E2, Palm Inc., Wokingham Berkshire, UK) to record information about each trial. The data Pendragon Forms mobile software was used to create a list of information to be recorded in a database for each trial. A second experimenter (the initiator) approached each subject from behind. The initiator placed herself with portable speakers (Travelsound 400, Creative, Dublin, Ireland or SP-106 Travel Speakers, Tesco Technika, Hertfordshire, England) and an mp3 (Zen Nomad Jukebox, Creative, Dublin, Ireland) player directly behind the subject at approximately 1 m distance. The height of the presentation was approximately 0.2–0.5 m below the ear level of the listener, so that the presentation could be done without being too conspicuous. Then the recorded sound was played back at a peak sound pressure level of 76.7 ± 3.9 dB re 20 μPa measured at a distance of 1 m (SPL level meter Rion NL-05, fast mode). If the subject did not respond immediately, the sound was produced again, up to a maximum of three times after which the trial was aborted if the subject had not responded. 62% of the participants responded in the first trial, 30% and the second, and 8% of the subjects in the third trial. There was no difference in turning biases in relation to the number of presentations (χ^2 ^= 0.83, N = 224, df = 2). Importantly, the initiator was instructed to playback sounds from a position directly behind the subject but was blind to the purpose of the study and also to the specific stimulus category played back in a given trial. Following each successful test, the subject was approached by both experimenters. The purpose of the experiment was explained and subject's handedness assessed. Further, the time spent living in the respective country was requested. People who had been living less than 10 years in the country were excluded from the analysis. The following information was recorded on the PalmOS handheld: the sound used or produced, the number of times played (up to a maximum of 3), the reaction orientation (right or left) for each subject, and the subject's gender and handedness. Since we obtained too few data for a meaningful analysis of left handed people, these data were discarded. The final sample consisted of 224 successful trials, each with a different individual. All people tested consented to the use of their data.

### Statistical Analysis

We used Binomial tests to examine orienting biases within the stimulus categories separately for each population (country). Results were corrected for multiple testing using a sequential Bonferroni correction (Step-Up Hochberg). In addition, a multinomial regression was used to test for the effects of country, stimulus category, and gender as well as the interactions between the main factors. All tests were calculated using SPSS 15.0.

### FMRI Experiment

#### Participants

22 right-handed, healthy young volunteers (10 female, aged from 22 to 34 years, mean age = 25.95 years) participated in the study. Handedness was assessed by the Edinburgh Handedness Inventory (Oldfield, 1971). Only subjects with normal hearing acuity were included. After being informed about potential risks and screened by a physician of the institution, subjects gave informed consent before participating. The experimental standards were approved by the local ethics committee of the University of Leipzig. Data were handled anonymously.

#### Stimuli

In addition to the stimuli described above (Speech and Artificial sounds), we also used time-reversed spoken words (Reverse) and non-linguistic human utterances as part of a separate investigation. In the Reverse class the same stimuli as in the Speech class were used but played-back in reverse. To further test whether hemispheric lateralization concerns species-specificity or acoustic features, we included a Human Sound class that comprised coughing, harrumphing, humming, and clicking one's tongue, i.e. four different non-linguistic species-specific sounds. Data from these conditions will not be considered further in the present paper. Half of the human utterances of all three classes were produced by two female speakers and the other half by two male speakers.

#### Presentation

**Figure 3:**
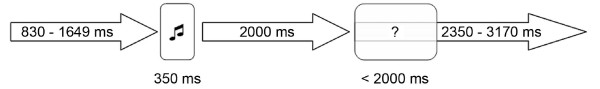
**Presentation of stimuli in the fMRI Experiment**. All trials followed the same temporal schema. Brain correlates were analyzed in an event-related design, time locked to stimulus onset. Performance was assessed by a forced choice after response cue presentation (question mark).

The experiment comprised of four conditions (Speech, Voice, Reverse, Artificial) and a visual control (Figure 3). Conditions were presented in randomized order (mixed trial design), and trial order differed for each subject. Within each trial of each condition, stimulation lasted 350 ms and was preceded by a variable gap (jittering) of either 830 ms, 1103 ms, 1376 ms, or 1649 ms. Stimulation was followed by a question mark presented after 2000 ms. This stimulus-onset-asynchrony was required in order to analyze brain activity without effects of the motor response. The question mark signalled the beginning of the response period which lasted 2000 ms at maximum; earlier responses aborted the question mark presentation. The next trial started after a variable interval (2350 to 3170 ms after the onset of the question mark, dependent on the jittering at the beginning of the trial). The trial-onset-asynchrony was 6 seconds. Except for the time during which the question mark was presented, the screen showed a fixation cross of 10 mm width and 10 mm height.

For all auditory conditions, sounds were presented as if coming from different locations in space. Five virtual sources were simulated by introducing different inter-aural time differences (ITDs): 0° (i.e. no ITD), 0.1 ms left (right) channel precedence (~10° deviance), or 0.2 ms left (right) channel precedence (~20° deviance). Within each auditory condition, the probability for a sound to be presented from 0° was 0.31; the probability for any other source was 0.167. In a visual control condition, a second cross was presented in addition to the original fixation cross 0.86° (sign centre) either to its right or to its left side. Stimulus presentation time was identical to that in the auditory conditions.

Three-hundred-twenty trials were presented overall, including 20 trials for the control condition and 20 empty trials (null-events). Among auditory conditions the number of trials differed such that hearing biological sounds (Speech, Voice, and Reverse) and non-biological sounds (Artificial) was equally probable, and, when hearing biological sounds, hearing linguistic (Speech) and non-linguistic (Voice, Reverse) sounds was equally probable, and when hearing non-linguistic sounds, hearing familiar/meaningful (Voice) and unfamiliar/meaningless (Reverse) sounds was equally probable. Accordingly, we presented 70 trials for Speech, 35 trials for Voice, 35 trials for Reverse, and 140 trials for Artificial.

#### Task Instructions

For the auditory conditions, participants were instructed to indicate whether they heard the stimulus coming rather from the right (pressing the right-hand button) or from the left (pressing the left-hand button). Participants were told that some stimuli were more difficult to locate than others, but they were naïve about the occurrence of stimuli coming from the 0° position. Participants were asked to deliver their response as fast as possible when the question mark appeared. For the visual control condition, participants were asked to indicate whether the second cross appeared left or right from the fixation cross by pressing the corresponding response button. Participants were instructed before the fMRI experiment.

#### Data Acquisition

In the MRI session, subjects were supine on the scanner bed with their right and left index finger positioned on the response buttons. In order to prevent postural adjustments, the subject's arms and hands were carefully stabilized by tape. In addition, form fitting cushions were used to prevent arm, hand and head motion. Participants were provided with earplugs (Bilsom model 303) to attenuate scanner noise (up to 41.8 dB). The auditory stimuli were presented via non-air tubes and through magnetic resonance-compatible electrostatic headphones ('Commander XG', Resonance Technology) which attenuates about 30 dB of gradient noise. Imaging was performed at 3T on a Bruker Medspec 30/100 system equipped with the standard bird cage head coil. Twelve axial slices (field of view 192 mm, 64 by 64 pixel matrix, thickness 3 mm, spacing 1 mm) parallel to the AC-PC plane were acquired using a single-shot gradient EPI sequence (TE = 30 ms, flip angle 90°, TR = 2000 ms) sensitive to BOLD contrast. Acquisition of the slices within the TR was arranged so that the slices were all rapidly acquired during 830 ms followed by a "silent" period of no acquisition (1170 ms) to complete the TR. This protocol, sometimes called bunched-early sequence, was used in order to present auditory stimuli without the gradient noise and so to enhance auditory perception. A set of 2D anatomical images was acquired for each subject immediately prior to the functional experiment, using a MDEFT sequence (256 × 256 pixel matrix). In a separate session, high resolution whole brain images were acquired from each subject to improve the localization of activation foci using a T1-weighted 3D segmented MDEFT sequence covering the whole brain.

#### Data Analysis

To assess whether responses to centrally presented stimuli differed in relation to the stimulus class, we calculated a repeated measures ANOVA of the proportion of right responses with stimulus as two-level within-subject factor (Speech, Artificial) and gender as between-subjects factor. The imaging data were processed using the software package LIPSIA [31]. This software package contains tools for pre-processing, co-registration, statistical evaluation, and visualization of fMRI data [see Additional file 4].

## Authors' contributions

JF and CT conceived the study, analyzed the behavioural data and wrote the manuscript, MD and AP conducted the behavioural experiments, JF, CT and RIS planned the fMRI experiments, RIS conducted the fMRI experiments and analyzed the fMRI data, DYC and RR provided technical expertise and logistical support. All authors commented on the manuscript.

## Supplementary Material

Additional file 1 **Additional behavioural experiment.** This file contains the procedure and results of an additional sound localization experiment. Click here for file

Additional file 2 **Speech samples.** The file contains examples of speech stimuli used in the experiments. The sampling frequency is 44.1 kHz. Click here for file

Additional file 3 **Artificial sound samples.** The file contains examples of artificial sound stimuli used in the experiments. The sampling frequency is 44.1 kHz. Click here for file

Additional file 4 **Analysis of fMRI data.** The file contains the specifics of the fMRI data analysis. Click here for file
