# Supplementary material for: Orienting asymmetries and lateralized processing of sounds in humans
Source: BMC Neurosci. 2009 Feb 24;10:14. doi: 10.1186/1471-2202-10-14 (PMC2652465; doi:10.1186/1471-2202-10-14)
Supplement: Additional file 1 — Additional behavioural experiment. This file contains the procedure and results of an additional sound localization experiment. [file 1471-2202-10-14-S1.doc]

### Additional behavioural experiment

To examine the influence of the experimental setting and instruction on orienting biases, we ran an experiment equivalent to the fMRI study in the free field azimuthal plane. Participants were 28 healthy, right-handed volunteers (14 female; aged from 20 to 30 years; mean age = 24.5 yrs.). Handedness was assessed by the Edinburgh Handedness Inventory (Oldfield, 1971). None of the subjects had participated in the fMRI experiment. Although it would have been desirable to recruit the same population of subjects, this was not feasible due to logistic reasons.

The participants were told that the aim of the study was to investigate their ability to localize sound sources and were thus ignorant as to the real objective of the study. Participants were instructed to focus on a fixation cross at the wall in front of them before each trial. They were asked to turn their head after hearing the sound as this would enhance their localization accuracy and then indicate as fast as possible whether the stimulus came from the right or from the left by pressing the respective buttons of a two-button response-box with their right or left index finger. After turning and indicating the position of the sound source, the participants were asked to return to their original position and focus on the cross. Participants were told that some stimuli were more difficult to locate than others, but they were naïve as to the occurrence of stimuli coming from the 0° position.

After the instructions, participants were seated in the middle of a soundproof chamber with their back against a semicircle of five loudspeakers hidden behind a black cloth. The loudspeakers were positioned 8° and 16° azimuth left and right of as well as 0° azimuth behind the place where the person was seated, at a distance of 2.3 m. The presentation of the stimuli was controlled from a neighbouring room. Each participant finished a session that lasted about 40 min without pause and comprised the presentation of 400 stimuli. The number of biological (Language, Reverse and Voice) and non-biological stimuli (Artificial) presented was equal. Within biological sounds, the number of linguistically meaningful sounds (Language) was equal to the number of non-linguistic sounds (Reverse and Voice), and within the latter class familiar (Voice) and unfamiliar non-linguistic sounds (Reverse) were presented equally often. In other words, we presented 200 Artificial sounds, 100 Language stimuli, and 50 stimuli of the Reverse and the Voice class, respectively. Within each stimulus class, the probability of each individual stimulus was equally high. Concerning the location of the sound source, 50% of the stimuli within each class were delivered from the central loudspeaker and 12.5% came from each of the remaining four speakers. The sequence of stimulus class was randomised. The first trial started as soon as the participant sat down in the right position and focused on the fixation cross at the wall. After orienting and indicating the position of the sound source, the participant turned back to the original position and focused on the cross again. Then the next stimulus was presented. The inter-stimulus interval varied randomly between 500 and 2000 ms (in 500 ms blocks). The average stimulus duration was 330 ± 135 ms (mean ± SD).

Subjects indicated the direction from which the sound was supposed to be coming by pushing one of two buttons. In addition, their head-turning behaviour was recorded by an observer concealed behind a one-way mirror. In 75 out of 11200 trials (0.66 %), subjects failed to produce one or either of the two responses; or they turned left but pushed the right button (or vice versa). These trials were excluded from further analysis. For statistical analysis, we calculated mean response rates and mean error rates, i.e., pressing/turning right (left) in response to a left (right) stimulus, per subject and condition. To assess whether responses to centrally presented stimuli differed in relation to the stimulus class, we calculated a repeated measures ANOVA of the proportion of right turns with Stimulus as four-level within-subject factor (Language, Voice, Reverse, Artificial). We analyzed the overall performance by comparing the error rates for the different conditions using a repeated-measures ANOVA (SPSS 14.0 GLM module) with the four-level factor Stimulus, the two-level factor presentation Side (right, left) and the two-level factor Deviance (10°, 20°, see below).

Responses to auditory stimuli presented from the 0° position were biased towards the right in all conditions (66.0% Language, 64.9% Voice, 66.0% Reverse, and 59.6% Artificial) which were all significantly different from chance (sign test; all P < 0.05). A repeated-measured ANOVA showed no main effect of STIMULUS on this bias (F(3, 24) = 2.19, p = 0.115), no influence of subject GENDER (p > 0.9) and no significant interaction STIMULUS × GENDER (p > 0.7). Error rates were influenced by the deviance and side from which sounds were presented. We found a significant interaction DEVIANCE × SIDE (F(1,26) = 93.2, p < 0.001), and a significant main effect for DEVIANCE (F(1,26) = 8.0, p < 0.009). The distribution of the data shows that sounds played from 8° from behind were more difficult to locate than when played from 16° (11.5% vs. 1.78% error rate). There were no significant main effects of STIMULUS (p > 0.8), SIDE (p > 0.09), or any other interactions (all p > 0.38). An inspection of the distribution of error rates revealed a tendency of attributing 8° left stimuli erroneously to the right side more frequently than 8° right stimuli to the left (16° right 0.7%, 8° right 8.4%, 8° left 14.7%, 16° left 2.8%, paired comparison between 8° left and 8° right: F = 5.8(1,27), p < 0.05), suggesting an overall right bias. This biased error pattern was not mirrored in reaction time data: there was no difference in RT in relation to which hand was used to press the button (mean ± SD RT: 1.33 ± 0.35 s R hand vs. 1.34 ± 35 s L hand for all responses with R (L) hand to sound played from R (L) or from the middle. Responses to central stimuli occurred later (1.47 ± 0.41 s) than those to presentations from 8° (1.34 ± 0.34 s) or 16° speakers (1.20 ± 0.32 s; F (4,24) = 15.6, p < 0.001).
