# Supplementary material for: Orienting asymmetries and lateralized processing of sounds in humans
Source: BMC Neurosci. 2009 Feb 24;10:14. doi: 10.1186/1471-2202-10-14 (PMC2652465; doi:10.1186/1471-2202-10-14)
Supplement: Additional file 4 — Analysis of fMRI data. The file contains the specifics of the fMRI data analysis. [file 1471-2202-10-14-S4.doc]

### Analysis of fMRI data

The LIPSIA software package was used for analysis of the imaging data [29]. Functional data were corrected for motion using a matching metric based on linear correlation. To correct for the temporal offset between the slices acquired in one scan, a sinc-interpolation based on the Nyquist-Shannon-Theorem was applied. A temporal highpass filter with a cutoff frequency of 1/72 Hz was used for baseline correction of the signal and a spatial Gaussian filter with 5.65 mm FWHM was applied. To align the functional data slices with a 3D stereotactic coordinate reference system, a rigid linear registration with six degrees of freedom (3 rotational, 3 translational) was performed. The rotational and translational parameters were acquired on the basis of the MDEFT [30,31] and EPI-T1 slices to achieve an optimal match between these slices and the individual 3D reference data set. This 3D reference data set was acquired for each subject during a previous scanning session. The MDEFT volume data set with 160 slices and 1mm slice thickness was standardized to the Talairach stereotactic space [32]. The rotational and translational parameters were subsequently transformed by linear scaling to a standard size. The resulting parameters were then used to transform the functional slices using trilinear interpolation, so that the resulting functional slices were aligned with the stereotactic coordinate system.

The statistical evaluation was based on a least-squares estimation using the general linear model for serially autocorrelated observations [33-35]. The design matrix was generated with a synthetic hemodynamic response function [36,37]. Brain activations were analyzed in an event-related design, timelocked to stimulus onset. The model equation, including the observation data, the design matrix and the error term, was convolved with a Gaussian kernel of dispersion of 4 s FWHM to deal with the temporal autocorrelation [35]. In the following, contrast-images, i.e. estimates of the raw-score differences between specified conditions, were generated for each participant.

The single-participant contrast-images were then entered into a second-level random effects analysis for each of the contrasts. The group analysis consisted of a one-sample t-test across the contrast images of all participants that indicated whether observed differences between conditions were significantly distinct from zero [38]. Subsequently, t-values were transformed into z-scores. To correct for false-positive results, in a first step, an initial voxelwise z-threshold was set to Z = 2.567 (p=.005, uncorrected). In a second step, the results were corrected for multiple comparisons using cluster-size and cluster-value thresholds obtained by Monte-Carlo simulations at a significance level of p=.005, i.e., the reported activations are significantly activated at p<.005, corrected for multiple comparisons at the cluster-level.

Contrast maps were generated that extracted the following effects of interest independently from each other; first, the main effect of localizing Speech sounds was investigated by building the contrasts between conditions Speech and Artificial. Second, the effect of localizing a 0° position sound was investigated by contrasting trials delivering a mid-position sound with trials delivering a right or left position sound. This contrast was build separately for both stimulus types (Speech and Artificial) in order to tap stimulus type specific activations.

References

29. Lohmann G, Müller K, Bosch V, Mentzel H, Hessler S, Chen L *et al*.: **LIPSIA--a new software system for the evaluation of functional magnetic resonance images of the human brain.** *Comp Med Imag Graphics* 2001, **25:** 449-457.

30. Ugurbil K, Garwood M, Ellermann J, Hendrich K, Hinke R, Hu X *et al*.: **Imaging at high magnetic fields: initial experiences at 4 T.** *Magn Reson Quart* 1993, **9:** 259-277.

31. Norris DG: **Reduced power multislice MDEFT imaging.** *Journal of Magnetic Resonance Imaging* 2000, **11:** 445-451.

32. Talairach J, Tournoux P: *Co-planar Stereotaxis Atlas of the Human Brain*. Stuttgart: Thieme; 1988.

33. Friston KJ: **Statistical parametric mapping.** In *Functional neuroimaging: technical foundation*. Edited by Thatcher RW, Hallet M, Zeffiro T, John ER, Huerta M. San Diega: Academic Press; 1994:77-93.

34. Friston KJ, Holmes AP, Poline JB, Grasby PJ, Willams SCR, Frackowiak RSJ *et al*.: **Analysis of fMRI time-series revisited.** *Neuroimage* 1995, **2:** 45-53.

35. Friston KJ, Holmes AP, Worsley KJ, Poline JB, Frith CD, Frackowiak RSJ: **Statistical parametric maps in functional imaging: a general linear approach.** *Human Brain Mapping* 1995, **2:** 189-210.

36. Friston KJ, Fletcher P, Josephs O, Holmes A, Rugg MD, Turner R: **Event-related fMRI: characterizing differential responses.** *Neuroimage* 1998, **7:** 30-40.

37. Josephs O, Turner R, Friston KJ: **Event-related fMRI.** *Human Brain Mapping* 1997, **5:** 243-248.

38. Holmes AP, Friston KJ: **Generalisability, random effects & population inference.** *Neuroimage* 1998, **7:** S754.
